# Deep learning‐based lung volume estimation with dynamic chest radiography

**DOI:** 10.1002/acm2.70487

**Published:** 2026-01-29

**Authors:** Nozomi Ishihara, Rie Tanaka, Haruto Kikuno, Noriyuki Ohkura, Isao Matsumoto

**Affiliations:** ^1^ College of Medical Pharmaceutical and Health Sciences, Kanazawa University Kanazawa Ishikawa Japan; ^2^ Department of Respiratory Medicine Kanazawa University Kanazawa Ishikawa Japan; ^3^ Department of Thoracic Surgery Kanazawa University Hospital Kanazawa Ishikawa Japan

**Keywords:** deep learning, dynamic chest radiography, lung volume

## Abstract

**Background:**

Dynamic chest radiography (DCR) is a recently developed low‐dose pulmonary functional imaging method that can be performed in a general X‐ray room. DCR provides sequential images during respiration, and the measured changes in lung area are a promising diagnostic indicator of lung function.

**Purpose:**

To investigate lung volume estimation using deep learning from DCR images during respiration and evaluate its accuracy in comparison with previously proposed estimation methods.

**Methods:**

Two convolutional neural networks (CNNs), VGG19 and DenseNet121, were trained using DCR image datasets from 257 patients, with reference lung volumes derived from corresponding computed tomography (CT) images. The performance of the models was evaluated using mean absolute error (MAE) and mean absolute percentage error (MAPE), and compared against that of a conventional linear regression model. Correlation between the estimated and reference lung volumes was assessed using Pearson's correlation coefficient (*r*) and the degrees‐of‐freedom‐adjusted coefficient of determination (*Rf^2^*). Forced vital capacity (FVC) was also estimated by subtracting the lung volume at maximum exhalation from that at maximum inhalation.

**Results:**

The VGG19 and DenseNet121 models demonstrated superior performance in estimating whole lung volume (combined right and left lung) compared to the linear regression method. Specifically, MAE was 373/376 mL, MAPE was 8.1%/7.9%, *r* was 0.88/0.90, and *Rf^2^* was 0.76/0.80 for VGG19/DenseNet121, respectively. In contrast, the linear regression model yielded an MAE of 568 mL, MAPE of 12.4%, *r* of 0.84, and *Rf^2^* of 0.69. Although the *Rf^2^* values for DCR‐derived FVC using VGG19 and DenseNet121 indicated moderate correlation, the MAE and MAPE were relatively high at 1.3/1.4 L and 41.1%/47.0%, respectively.

**Conclusion:**

The proposed deep learning‐based approach for lung volume estimation from DCR images outperformed the conventional linear regression method. Further improvements in CNN model architecture and the incorporation of guided forced respiratory maneuvers may enhance the potential for image‐based pulmonary function testing.

## 1 INTRODUCTION

Respiratory diseases, including chronic obstructive pulmonary disease (COPD), lower respiratory tract infections, and cancers of the trachea, bronchus, and lungs, are among the leading causes of death worldwide.^1^ Lung dysfunction associated with these conditions often manifests as changes in lung volume, which are typically assessed using pulmonary function tests (PFTs) for diagnostic and severity evaluation. However, some patients may experience difficulty performing forced respiration using a mouthpiece or nosepiece, hindering accurate pulmonary function assessment. Additionally, because PFTs involve oral examination, they are generally contraindicated in patients with infectious diseases to prevent the risk of transmission through contaminated equipment.^2^ These limitations have led to increased interest in imaging‐based approaches to pulmonary function assessment.

Pulmonary functional imaging is currently available through several modalities, including nuclear medicine,^3^, ^4^ computed tomography (CT),^5^, ^6^, ^7^ and magnetic resonance imaging (MRI).^8^, ^9^ These techniques provide a wide range of functional data, including lung volume changes, regional ventilation and perfusion, and morphological characteristics. However, their clinical utility is limited due to factors such as long examination times, procedural complexity, and high costs. In response to these challenges, a novel method known as dynamic chest radiography (DCR), utilizing a flat‐panel detector (FPD) in a standard X‐ray room, has been developed.^10^

DCR captures sequential images during respiration at radiation doses comparable to those of conventional chest radiography (CXR) and enables assessment of lung function through changes in lung density, lung area, and diaphragm position during breathing; reduced ventilatory and perfusion areas are detected as regions with diminished changes.^11^, ^12^, ^13^, ^14^, ^15^, ^16^, ^17^, ^18^, ^19^, ^20^ Studies have shown that the lung area observed in frontal DCR images correlates with forced expiratory volume in one second (FEV_1_) and FEV_1_%.^21^ Moreover, the rate of change in lung area is associated with respiratory function parameters such as residual volume/total lung capacity (RV/TLC), FEV_1_%, and COPD assessment scores.^22^, ^23^ However, these studies were limited by small sample sizes or patient cohorts restricted to specific diseases, and they did not provide direct, quantitative lung volume measurements. As lung volume is a key biomarker of pulmonary function, directly estimating right and left lung volumes from DCR could offer more comprehensive insights into respiratory health. Therefore, this study focuses on estimating right and left lung volumes using DCR images.

Several prior studies have addressed lung volume estimation from DCR images. Ambrose et al. used Pratt's method^24^ to estimate lung volume based on the lung area from frontal and lateral DCR images of 15 patients during deep breathing and demonstrated correlation with TLC obtained through whole‐body plethysmography.^25^ Ueyama et al. estimated forced vital capacity (FVC), TLC, functional residual capacity (FRC), and RV in 97 patients with interstitial lung disease using lung volumes derived from frontal and lateral DCR images, in combination with age, sex, and body mass index (BMI).^19^ For TLC, the reported adjusted *R^2^*, root mean square error (RMSE), mean absolute error (MAE), and correlation coefficient (*r*) were 0.813, 410 mL, 328 mL, and 0.91, respectively. Additionally, FitzMaurice et al. developed a linear regression model to estimate lung volume using segmented lung area from frontal DCR images and patient height in 20 patients with cystic fibrosis, achieving a multiple *R^2^* of 0.77 and an adjusted *R^2^* of 0.75 (*P* < 0.001).^26^ Despite these promising results, existing methods have several limitations: small sample sizes, lack of validation or control groups, the need for manual lung area tracing, added radiation exposure from lateral imaging, and applicability limited to specific diseases. In contrast, recent studies have applied deep learning to estimate lung volumes from CXR images using large datasets that include abnormalities such as COPD and lung nodules.^27^, ^28^ One such model, trained on real frontal CXR images with PFT‐derived labels, achieved an MAE of 509 mL and a mean absolute percentage error (MAPE) of 10.3%.^27^ Applying deep learning to DCR has the potential to overcome the limitations of manual processing and segmentation, enabling efficient and accurate lung volume estimation using only frontal images. Given that high estimation accuracy has already been achieved using frontal CXR images alone,^27^ this study aimed to estimate right and left lung volume from frontal DCR images to minimize radiation exposure. Therefore, the objective of this study was to investigate deep learning‐based estimation of right and left lung volume from frontal DCR images and to evaluate its accuracy in comparison with previously proposed estimation methods.

## 2 MATERIAL AND METHODS

### 2.1 Study design

A DCR image acquired at the same inspiratory level as the corresponding chest CT image was selected from among multiple respiratory‐phase DCR images. The selection was based on matching the lung area with that of a digitally reconstructed radiograph (DRR) generated from the CT image of the same patient. Two convolutional neural networks (CNNs) were then trained using the selected DCR images and the reference lung volumes derived from the CT images. The performance of the CNN models was evaluated by assessing the agreement between the lung volumes estimated from the DCR images and the reference volumes obtained from CT images.

### 2.2 Study population

This study included 337 patients who underwent chest CT as part of routine clinical care, selected from a total of 697 patients with respiratory diseases who underwent DCR at XXXX between December 2015 and February 2021. The exclusion criteria were as follows: postoperative anatomical changes (*n* = 13); cardiogenic reversal (*n* = 1); and imaging findings such as severe interstitial pneumonia, pleural effusion, or tumors that obscured clear identification of the lung areas on CT or DCR images (*n* = 33). Additionally, 33 cases were excluded due to the absence of compatible images between DCR and DRR caused by mismatched respiratory phases. As a result, 257 patients (age range: 35–88 years; mean age: 68 years; male‐to‐female ratio: 176:81) were included in the final analysis. Patient characteristics are summarized in Table 1. This study was approved by the Medical Ethics Review Committee of Kanazawa University (registration number: 114188‐2), and written informed consent was obtained from all participants.

**TABLE 1:** Patient characteristics.

Number	257
Age (years)	68.4 ± 9.5
Sex (female/male)	81/176
Height (cm)	162.3 ± 9.0
Weight (kg)	59.6 ± 10.8
BMI (kg/m^2^)	22.6 ± 3.4
Classification of respiratory diseases	
Lung cancer	153
ILD	42
COPD	31
BA	8
ACO	5
Others	18

BMI: body mass index; ILD: interstitial lung disease; COPD: chronic obstructive pulmonary disease; BA: bronchial asthma; ACO: asthma and COPD overlap

### 2.3 Acquisition of DCR images

Sequential images were acquired using an X‐ray imaging system (Test Model; Konica Minolta, Tokyo, Japan) equipped with an indirect FPD (PaxScan 4343CB; Varex Imaging Corp., Utah, USA) and an X‐ray generator/tube (DHF‐155H II/UH‐6QC‐07E; Hitachi Healthcare, Ltd., Tokyo, Japan). Imaging was performed in the posteroanterior direction during forced respiration with the patient in a standing position, using pulsed irradiation at 15 frames/s (100 kV, 0.2 mAs/pulse, source‐to‐detector distance = 2.0 m, 0.2 mm Cu additional filter), according to the standard imaging conditions developed for DCR.^10^ An automated voice guidance system instructed patients to ensure that a complete respiratory cycle was captured within approximately 14 s of imaging time. The average number of images acquired per examination was 233.9 ± 11.8, corresponding to a mean total tube current–time product of 46.8 ± 2.4 mAs. The total entrance surface dose was kept below the guidance level for two projections (1.9 mGy) as recommended by the International Atomic Energy Agency.^29^, ^30^ The resulting DCR images had a matrix size of 1024 × 1024 pixels, a pixel size of 417 × 417 µm^2^, and a 16‐bit grayscale.

### 2.4 Calculation of lung volume from the CT images

Chest CT images served as the gold standard for lung volume measurement. CT scans were performed at end‐inspiration using two scanners: a 256‐slice CT scanner (Revolution CT; GE Healthcare, Milwaukee, WI, USA) and a dual‐source CT scanner (SOMATOM Definition Flash; Siemens Healthineers, Erlangen, Germany). All scans were acquired in helical mode at 120 kV, with current modulation (Smart mA for GE; CareDose 4D for Siemens) applied to reduce radiation exposure. The rotation time was 0.35 s for GE and 0.5 s for Siemens, with a pitch of 0.992 and 0.9, respectively. Detector configurations were 128 × 0.625 mm (GE) and 64 × 0.6 mm (Siemens). Images were reconstructed with a section thickness of 0.625–1.0 mm, an interval of 0.5–0.7 mm, and a high‐spatial‐frequency kernel (Lung or Bone Plus for GE; B75f for Siemens). All images covered the entire lung field with a 512 × 512 matrix and 16‐bit grayscale.

All CT images were resized to 256 × 256 pixels in 8‐bit portable network graphics (PNG) format and processed using a U‐Net‐based lung segmentation model previously developed on a separate CT dataset in our earlier study (Dice coefficient = 0.98) (Neural Network Console version 2.1.7790.33921, Sony Network Communications Inc).^31^, ^32^ The lung volumes were calculated based on the number of pixels in the lung field, pixel size, and slice thickness of the CT images.

### 2.5 Selection of the DCR image at the same respiratory level as the CT image

**FIGURE 1:**
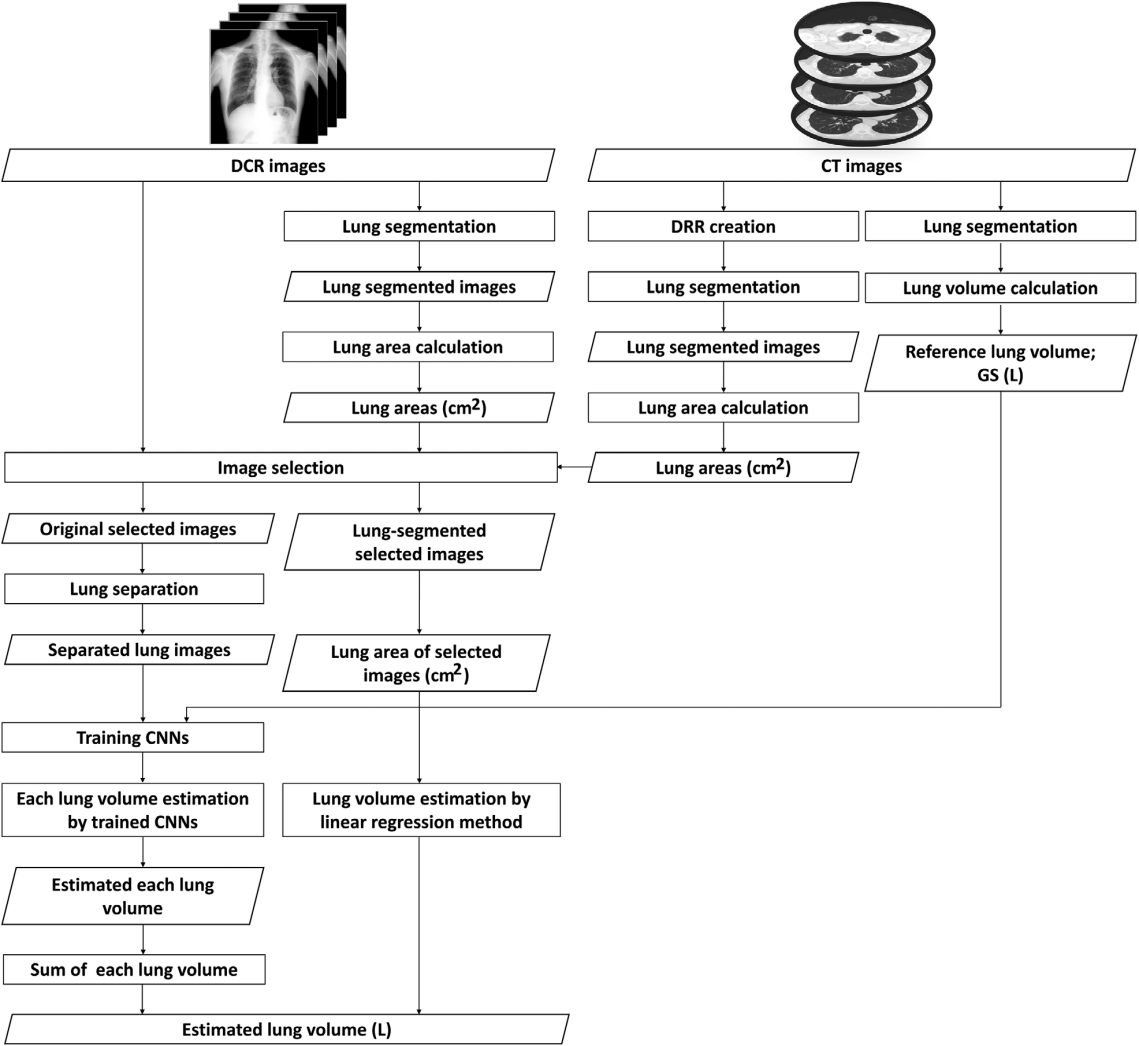
Overall scheme of the image‐processing process. The rectangles, rhombus, and arrows represent the procedure, data, and data processing flow, respectively. DCR: dynamic chest radiography, CT: computed tomography; DRR: digitally reconstructed radiography; GS: gold standard; CNNs: convolutional neural networks.

Figure 1 illustrates the overall image processing workflow. The CT images were first resampled to isotropic voxels based on their respective pixel sizes, then averaged along the coronal plane from a virtual point‐source projection to generate a DRR resembling a conventional chest radiograph. Magnification effects due to patient positioning within the CT volume were inherently preserved, and no additional centering or geometric correction was applied prior to DRR generation. Both the DRR and DCR images were resized to 256 × 256 pixels in 8‐bit PNG format. Lung regions were segmented using the U‐Net‐based model developed in our previous study (Dice coefficient = 0.97) (Neural Network Console version 2.1.7790.33921, Sony Network Communications Inc.).^31^, ^32^ The DCR image with the lung area most closely matching that of the DRR image was selected (see Supplementary Figure S1 for an illustration of the workflow). This selected DCR image was then divided into right and left lung regions to enable individual lung volume estimation.

### 2.6 Lung volume estimation using deep learning

Two deep learning models—VGG19^33^ and DenseNet121^34^—were implemented for lung volume estimation. These architectures have previously demonstrated high accuracy in estimating lung volumes from CXR images.^27^ Each network was designed to output a single regression value representing the estimated lung volume in liters.

To develop the regression models, the dataset was divided into three subsets: training (*n* = 206), validation (*n* = 26), and testing (*n* = 25), in an 8:1:1 ratio. Data augmentation was applied to the training and validation datasets to enhance model generalization. Augmentation techniques included variations in window level (±50) and width (±50), rotation (±5°), and horizontal (±20 pixels) and vertical translations (+15, −5 pixels). Following augmentation, the total number of images increased from 232 to 2552.

Each model was trained by minimizing the mean squared error (MSE) between the predicted and reference lung volumes, using the Adam optimizer. The learning rate, batch size, and number of training epochs were optimized and set to 0.001, 32, and 1000, respectively. Training and testing were conducted in the following environment: CPU—Intel Core i9‐10900K @ 3.70 GHz; GPU—NVIDIA GeForce RTX 3090; Programming language—Python 3.8.13; Framework—PyTorch 1.8.2.

All pre‐trained weights were reinitialized, allowing the models to learn entirely from scratch in this study (see Supplementary Figure S2 for the training/validation curves).

### 2.7 Lung volume estimation using the liner regression method

Lung volumes were also calculated using FitzMaurice's method (hereafter known as the linear regression method) based on the projected lung area at the inspiratory phase and patient height, which was developed to calculate TLC in patients with cystic fibrosis^26^:

$$\begin{array}{cc} TLC\;\;\left(L\right) & =Projected\;lung\;area_{insp}\;\left(cm^{2}\right)\times 0.008\\ & \;+height\;\left(cm\right)\times 0.079-10.7 \end{array}$$

### 2.8 Statistical analysis

To evaluate the performance of the developed CNN models for lung volume estimation, the following metrics were calculated: error (defined as estimated lung volume—reference lung volume), MAE (= Σ |error|/number of patients), and MAPE (= Σ |error/reference lung volume|/number of patients × 100). In addition, Pearson's correlation coefficient (*r*) and the coefficient of determination (*R^2^*) from linear regression were used to assess the correlation between estimated and reference lung volumes. The 95% confidence intervals (CI) for Pearson's *r* were also calculated.

Given the relatively small sample size, the *R^2^* values were adjusted to degrees‐of‐freedom‐corrected coefficients of determination (*R_f_^2^*), using the formula:

$${R_{f}}^{2}=1-\left(1-R^{2}\right)\times \frac{N-1}{N-P-1}$$

where *N* is the sample size, *P* is the number of explanatory variables in the model, and *N*–*P*–*1* indicates the degrees of freedom. Based on established criteria,^35^ coefficients of determination were interpreted as follows: *R_f_^2^* ≤ 0.12 indicated a weak correlation, 0.12 < *R_f_^2^* < 0.45 indicated a moderate correlation, and *R_f_^2^* ≥ 0.45 indicated a strong correlation. An *R_f_^2^* > 0.7 was considered to represent a high correlation.^36^

Furthermore, a paired *t*‐test was conducted to evaluate whether there was a statistically significant difference between the lung volumes estimated by the VGG19 and DenseNet121 models. A *P*‐value < 0.01 was considered statistically significant.

### 2.9 FVC estimation

Respiratory changes in lung volume corresponding to FVC in PFT were derived using the developed models (hereafter referred to as DCR‐derived FVC). DCR‐derived FVC was calculated by subtracting the lung volume estimated at maximum exhalation from that at maximum inhalation using the DCR images of 25 test patients. The accuracy of DCR‐derived FVC was evaluated using the MAE and MAPE with the PFT‐derived FVC serving as the reference.

Correlation between the PFT‐derived and DCR‐derived FVC values was assessed using *r* and *R_f_^2^*. Additionally, a paired *t*‐test was conducted to determine whether there was a statistically significant difference between the FVC values estimated by the VGG19 and DenseNet121 models. A *P*‐value < 0.01 was considered statistically significant.

## 3 RESULTS

The lung volumes estimated using the CNN models and the conventional linear regression method are summarized in Table 2. For training with the right and left lung images separately, the MAE and MAPE were 222/210 mL and 8.8%/9.8% for the VGG19 model, and 239/201 mL and 9.0%/9.6% for the DenseNet121 model, respectively. When combining the right and left lung volumes to calculate the whole lung volume, the MAE and MAPE were 373 mL and 8.1% for the VGG19 model, and 376 mL and 7.9% for the DenseNet121 model.

**TABLE 2:** Estimation performance of each method.

		MAE [mL]	MAPE [%]	Pearson's *r*	*R_f_^2^*
CNN models	VGG19				
	Right lung	222	8.8	0.84	0.70
	Left lung	210	9.8	0.85	0.70
	Whole lung (combined right and left lung)	373	8.1	0.88	0.76
	DenseNet121				
	Right lung	239	9.0	0.85	0.70
	Left lung	201	9.6	0.84	0.69
	Whole lung (combined right and left lung)	376	7.9	0.90	0.80
Linear regression method	The linear regression method	568	12.4	0.84	0.69

CNN: convolutional neural network; MAE: mean absolute error; MAPE: mean absolute percentage error; *R_f_^2^*: degrees of freedom‐adjusted coefficients of determination

**FIGURE 2:**
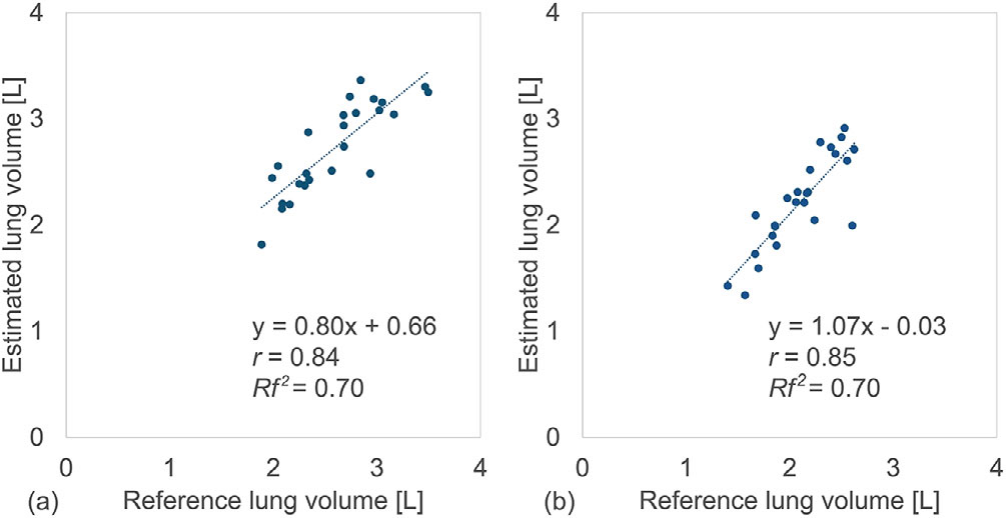
Relationship between the reference and estimated right and left lung volumes of 25 patients obtained using convolutional neural network (CNN)‐based method: VGG19 model in (a) right and (b) left lungs, respectively.

**FIGURE 3:**
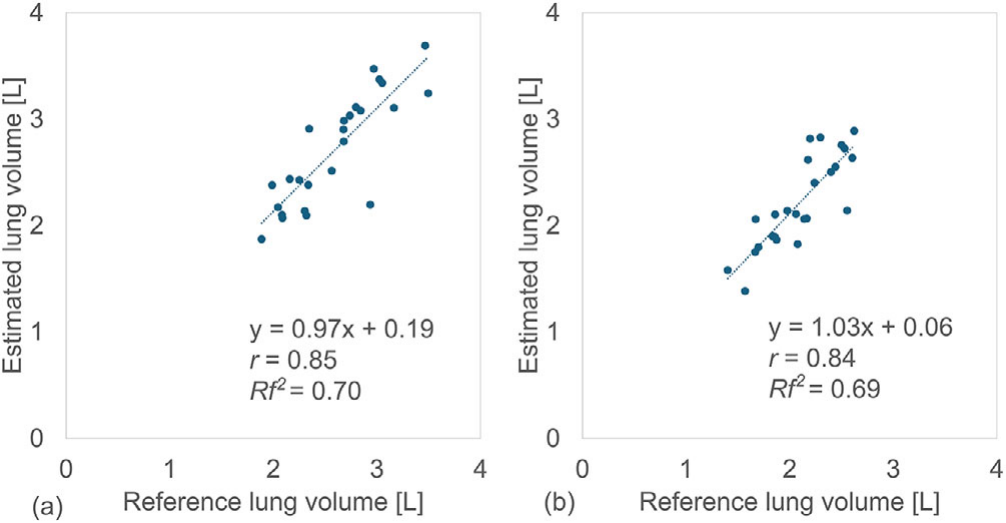
Relationship between the reference and estimated right and left lung volumes of 25 patients obtained using convolutional neural network (CNN)‐based method: DenseNet121 model in (a) right and (b) left lungs, respectively.

**FIGURE 4:**
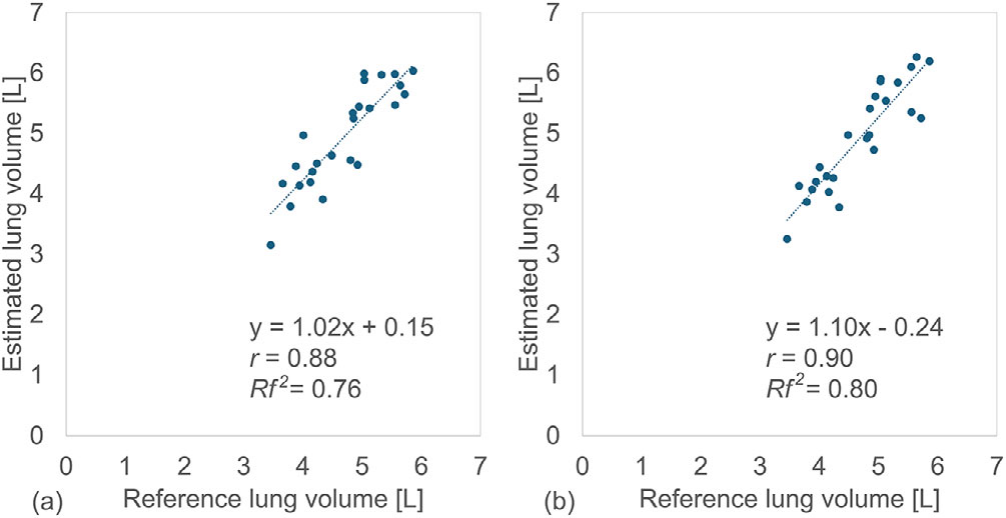
Relationship between the reference and estimated whole lung volumes calculated by combining the right and left lung volumes of 25 patients obtained using convolutional neural network (CNN)‐based methods: (a) VGG19 and (b) DenseNet121, respectively.

**FIGURE 5:**
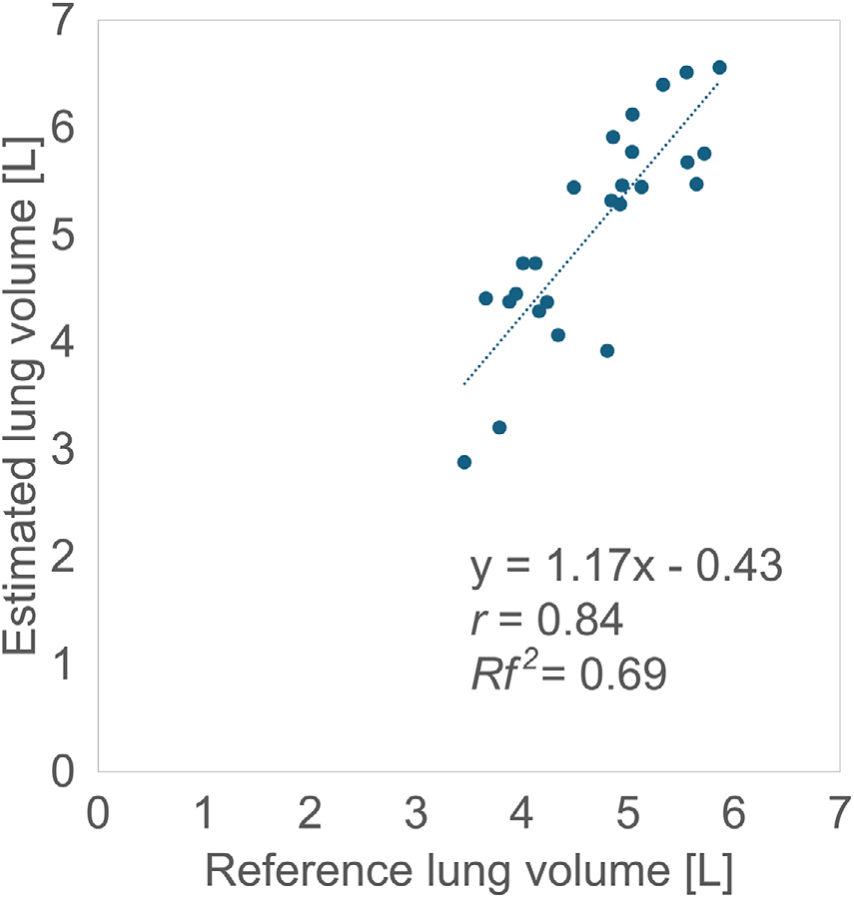
Relationship between the reference and estimated lung volumes using the linear regression method.

The CNN models demonstrated strong linear correlations between the reference and estimated lung volumes from the right/left lung images. For the VGG19 model, the *r* values were 0.84/0.85 (95% CI: 0.67–0.93/0.68–0.93), and *Rf^2^* values were 0.70/0.70 (Figure 2). Similarly, for the DenseNet121 model, the *r* values were 0.85/0.84 (95% CI: 0.68–0.93/0.66–0.93), and *Rf^2^* values were 0.70/0.69 (Figure 3). For whole lung volume estimation, the *r* and *Rf^2^* values were 0.88 (95% CI: 0.74–0.95) and 0.76 for the VGG19 model, and 0.90 (95% CI: 0.79–0.96) and 0.80 for the DenseNet121 model (Figure 4). No statistically significant differences were observed between the VGG19 and DenseNet121 models for right/left or whole lung volumes (*P* > 0.01). In contrast, the conventional linear regression method showed an MAE and MAPE of 568 mL and 12.4%, respectively (Table 2). The correlation between reference and estimated lung volumes using linear regression yielded an *r* of 0.84 (95% CI: 0.67–0.93) and an *Rf^2^* of 0.69 (Figure 5).

**FIGURE 6:**
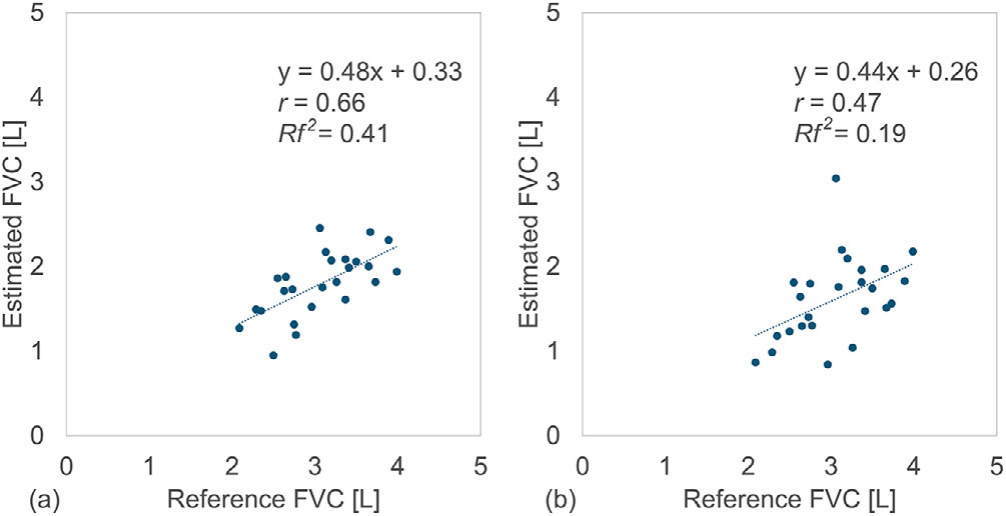
Relationship between the reference and estimated forced vital capacity (FVC) of patients obtained using convolutional neural network (CNN)‐based methods: (a) VGG19 and (b) DenseNet121 model, respectively.

For DCR‐derived FVC estimation, the MAE and MAPE were 1.3 L and 41.1% for the VGG19 model, and 1.4 L and 47.0% for the DenseNet121 model. The corresponding *r* and *Rf^2^* values were 0.66 (95% CI: 0.36–0.84) and 0.41 for the VGG19 model, and 0.47 (95% CI: 0.09–0.73) and 0.19 for the DenseNet121 model (Figure 6). Again, no statistically significant difference in FVC was found between the two models (*P* > 0.01).

## 4 DISCUSSION

In this study, two CNN models—VGG19 and DenseNet121—were trained on DCR images from 206 patients to estimate right and left lung volumes, with the summed whole lung volumes compared against a conventional linear regression method. For whole lung volume estimation, the MAE and MAPE were 373 mL and 8.1% for VGG19, 376 mL and 7.9% for DenseNet121, and 568 mL and 12.4% for the linear regression method. Additionally, both CNN models demonstrated higher *r* and *Rf^2^* than the linear regression approach.

For DCR‐derived FVC, *Rf^2^* values indicated moderate correlations for both CNN models (VGG19/DenseNet121), while the MAE and MAPE were 1.3/1.4 L and 41.1%/47.0%, respectively. Although further improvement is necessary for the clinical application of DCR‐derived FVC, this study demonstrates the feasibility of deep learning‐based lung volume estimation from DCR images.

The lower MAE values observed for the CNN models compared to the linear regression method suggest that deep learning‐based approaches are more effective for estimating lung volumes from frontal DCR images, and that a linear regression model derived from a disease‐specific cohort may have limited clinical applicability. In addition, our trained CNNs were better or comparable to those developed in previous studies: the MAE was 328 mL for Ueyama's method, which used frontal and lateral DCR in combination with age, sex, and BMI,^19^ 650 mL for frontal CXR, and 408 mL for frontal and lateral CXR with deep learning.^27^ Although the performance of lung volume estimation using frontal and lateral DCR with the linear regression method was better than that of our trained CNNs, it was developed using only 97 patients with interstitial lung disease and validated using the same patient group.^19^ In contrast, our training dataset included 206 patients with various respiratory diseases—such as lung cancer, interstitial lung disease, COPD, bronchial asthma, asthma‐COPD overlap, and others—indicating high generalizability. However, the accuracy of FVC estimation was lower than that reported in a previous study (MAE; 272 mL, *Rf^2^*; 0.814), in which lung volumes were calculated using frontal and lateral DCR in both expiratory and inspiratory phases.^19^ In contrast, our study used only the maximal inspiratory phase for training, which may have resulted in less accurate estimations of expiratory‐phase lung volume.

This study had several limitations. First, the reference lung volumes were calculated from CT images in the supine position, whereas DCR was performed in the upright position. A previous study reported that lung volume differs by approximately 10% between standing and supine positions,^37^ suggesting that this positional mismatch may have increased the estimation error. Second, the CNN models were trained on a single DCR image acquired at the same respiratory level as the inspiratory CT image, rather than on DCR images covering multiple respiratory phases, including maximal expiration. To address this, future models should be trained using sequential DCR images with known lung volumes, such as those generated from realistic digital phantoms.^38^, ^39^, ^40^ Third, the breathing pattern during DCR imaging may not have involved forced respiration, which probably caused large MAE and MAPE errors in FVC estimation. Aligning the breathing instructions for DCR and PFT could help bring DCR‐derived FVC values closer to those obtained from PFT. Finally, this study was conducted using data from a single institution, which may have introduced biases in the dataset, such as a small number of patients with respiratory diseases in each group and the absence of a healthy control group. Future research with a larger and more diverse cohort is needed to improve estimation accuracy.

Despite its limitations, deep learning‐based lung volume estimation using DCR has several advantages over conventional PFT. Notably, DCR provides additional morphological information for each lung. While PFT assesses total lung volume, DCR estimates right and left lung volumes separately and also derives PFT‐like parameters such as FVC, which can aid in post‐lobectomy follow‐up and the assessment of unilateral lung disease. Additionally, DCR provides functional data beyond volume, such as regional ventilation and perfusion, inferred from changes in lung density during respiration.^10^ This combination of anatomical and functional information may allow simpler and more comprehensive lung function evaluation than PFT alone. Furthermore, DCR is a non‐oral examination, allowing examiners to maintain physical distance from patients and reducing the risk of infection—an advantage during infectious disease outbreaks. The deep learning‐based approach also eliminates the need for manual and time‐consuming operations required in regression‐based volume estimation. Together, DCR and deep learning could contribute to more accessible and adaptable pulmonary function assessments suited to various clinical and societal contexts.

The DCR system became commercially available in 2018 and was approved by the U.S. Food and Drug Administration in 2019. As accessibility has increased, DCR can now be added to standard radiography protocols. Although DCR involves approximately twice the radiation exposure of CXR, it remains acceptable due to its high diagnostic yield and efficient imaging workflow, supporting its use in routine clinical practice. While certain challenges remain, this study demonstrated the feasibility of deep learning‐based lung volume estimation using DCR.

## 5 CONCLUSION

The developed deep learning‐based lung volume estimation models—VGG19 and DenseNet121—demonstrated higher estimation accuracy (MAE of 373/376 mL, MAPE of 8.1%/7.9%, *r* of 0.88/0.90, and *R_f_^2^* of 0.76/0.80, respectively) compared to the linear regression method. Although there remains room for improvement in both the trained CNN models and the respiratory guidance during DCR imaging, this study confirmed the feasibility of deep learning‐based right and left lung volume estimation from DCR images. Continued development in this area may lead to the establishment of imaging‐based routine pulmonary function evaluation.

## AUTHOR CONTRIBUTIONS

All authors made substantial contributions to all of the following: (1) the conception and design of the study, or acquisition of data, or analysis and interpretation of data, (2) drafting the article or revising it critically for important intellectual content, (3) final approval of the version to be submitted.

## ETHICAL APPROVAL

This study was approved by the Medical Ethics Review Committee of Kanazawa University (registration number: 114188‐2), and written informed consent was obtained from all participants.

## CONFLICT OF INTEREST STATEMENT

The authors declare no conflicts of interest.

Our institution received a research grant from Konica Minolta, Inc., Tokyo, Japan.

## Supporting information



Supporting Information

Supporting Information
