# Supplementary material for: Deep learning‐based lung volume estimation with dynamic chest radiography
Source: J Appl Clin Med Phys. 2026 Jan 29;27(2):e70487. doi: 10.1002/acm2.70487 (PMC12854853; doi:10.1002/acm2.70487)

Illustration of the automatic matching process based on lung area between dynamic chest radiography (DCR) and a digitally reconstructed radiograph (DRR) generated from the corresponding CT image. The matched lung area frame was automatically selected.

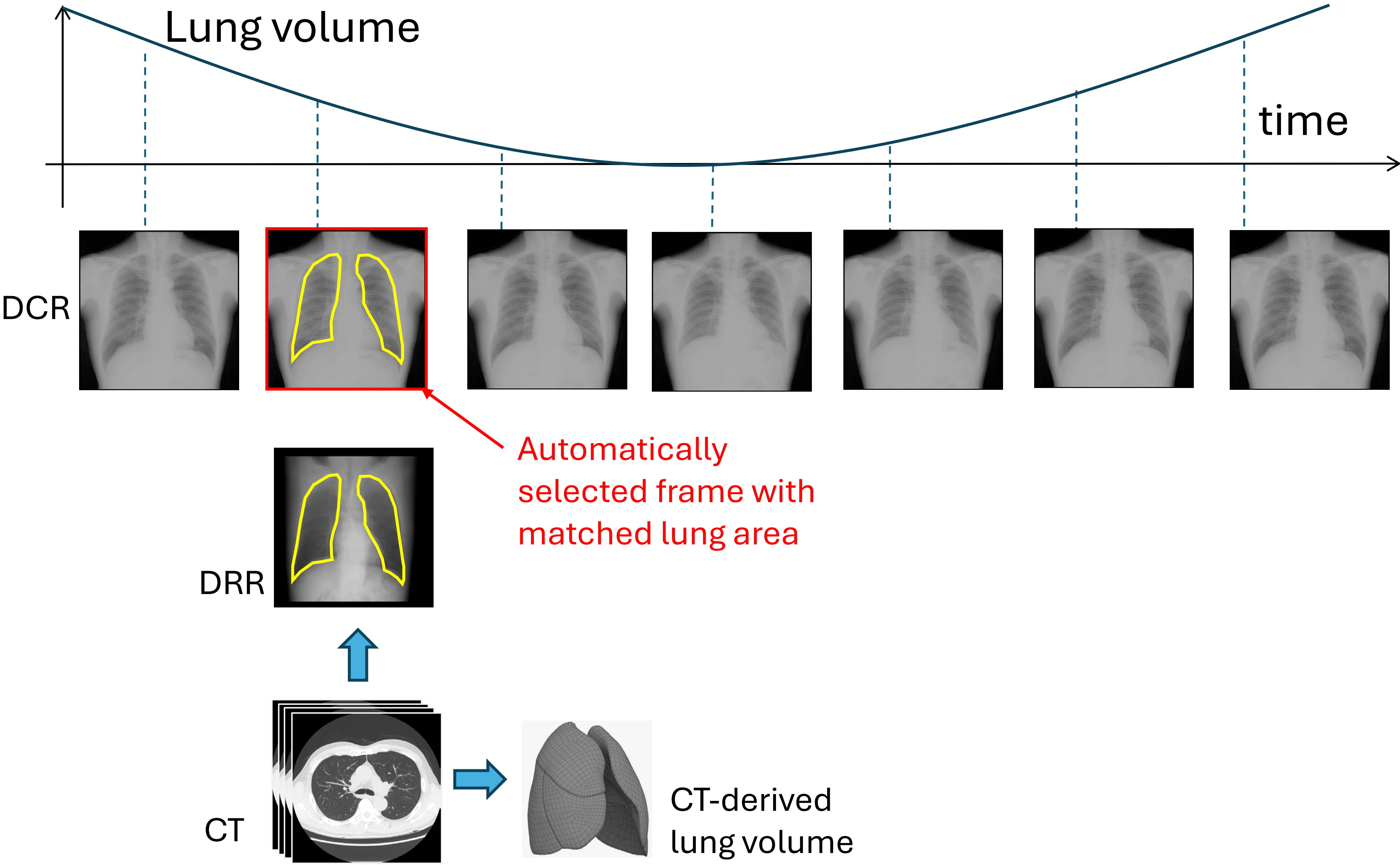

Supplement: Supplementary file 1 — Supporting Information [file ACM2-27-e70487-s002.pdf]
