# Supplementary material for: Deep learning‐based lung volume estimation with dynamic chest radiography
Source: J Appl Clin Med Phys. 2026 Jan 29;27(2):e70487. doi: 10.1002/acm2.70487 (PMC12854853; doi:10.1002/acm2.70487)

Training and validation learning curves of DenseNet121 (top row) and VGG19 (bottom row) for the right and left lung models

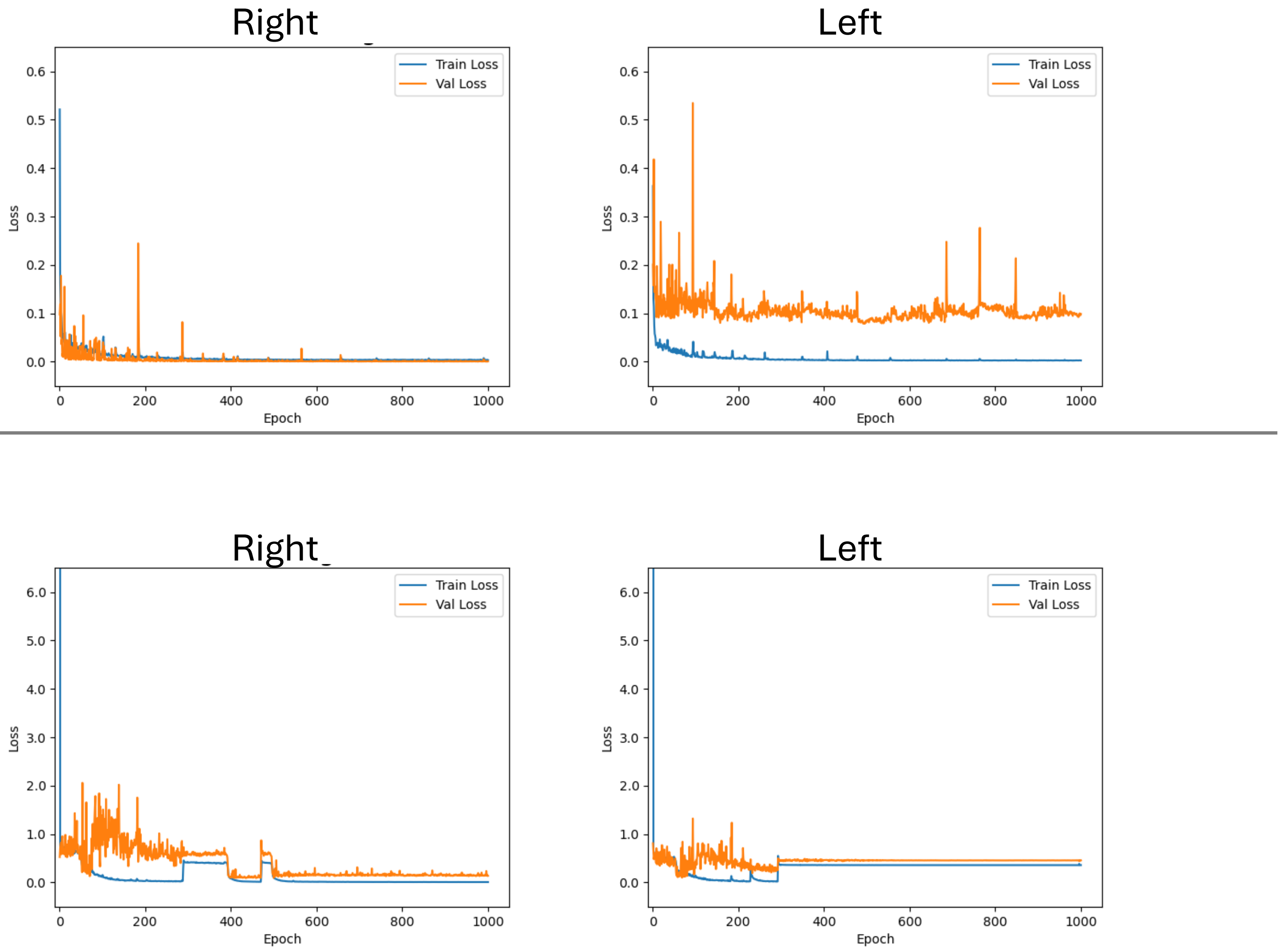

Supplement: Supplementary file 2 — Supporting Information [file ACM2-27-e70487-s001.pdf]
